# Development and Validation of a Test for the Classification of Horses as Broken or Unbroken

**DOI:** 10.3390/ani11082303

**Published:** 2021-08-04

**Authors:** Laura Menchetti, Emanuela Dalla Costa, Michela Minero, Barbara Padalino

**Affiliations:** 1 Department of Agricultural and Food Sciences, Alma Mater Studiorum–University of Bologna, 40127 Bologna, Italy; laura.menchetti@unibo.it; 2 Department of Veterinary Medicine, University of Milan, 26900 Lodi, Italy; emanuela.dallacosta@unimi.it (E.D.C.); michela.minero@unimi.it (M.M.)

**Keywords:** transport, equine, Broken/Unbroken Test (BUT), validity, reliability, welfare

## Abstract

**Simple Summary:**

Transportation is a stressful event for all animal species, but some species may be subjected to worse welfare consequences than others due to their ethological characteristics and specific coping strategies. Among equines, horses with a low level of tameness are at higher risk for transport-related disease and injury. For this reason, in Europe, regulations for the protection of animals during transport (Regulation EC 1/2005) are stricter for unbroken (untamed) vs. broken (tamed) horses. However, in practice, official veterinarians cannot verify regulatory compliance as there is no valid tool for the classification of horses as broken or unbroken. This study proposes the Broken/Unbroken Test (BUT) for assessment and scoring of horse behaviour during approach, haltering, and handling. After a validation process, our study has shown that the BUT is a reliable, valid, and feasible tool for determining whether a horse is broken or unbroken. The use of this tool would allow simple verification of compliance with Regulation 1/2005, and would help to ensure that transport procedures for unbroken horses are more respectful of their ethological and physiological characteristics. This may reduce the incidence of adverse welfare consequences for horses during transportation.

**Abstract:**

Regulation EC 1/2005 has stricter rules for transportation of unbroken (untamed) vs. broken (tamed) horses, but does not provide adequate tools for their identification. This study aimed to develop and validate such a tool. A behavioural test (Broken/Unbroken Test (BUT)) based on approaching, haltering, and leading was applied to 100 horses. Physiological and additional behavioural data were also collected, and the horses’ status (broken/unbroken) was assessed by the expert who administered the BUT. Each horse’s behaviour during the BUT was scored by four trained observers blinded to the horse’s history. The BUT score showed excellent inter-observer, intra-observer, and test–retest reliability (all intraclass correlation coefficients (ICCs) > 0.75). It was also negatively associated with respiratory rate, avoidance distance, and time needed to approach, halter, and lead the horse (*p* < 0.05 for all). The optimal BUT score cut-off for discrimination between broken and unbroken horses (gold standard: expert judgment) showed 97.8% sensitivity and 97.3% specificity. There was almost perfect agreement between BUT-based and expert classification of horses (ICC = 0.940). These findings confirm the BUT’s construct and criterion validity. The BUT could provide officials with a feasible, reliable, and valid tool to identify a horse’s broken/unbroken status and, consequently, direct stakeholders towards correct transport procedures.

## 1. Introduction

Every year, millions of horses are transported over long distances by road, sea, and air [1,2]. Horses may be transported for various purposes and, unlike other farmed species, many times in their lives [3], and travel conditions and related welfare consequences differ depending on the situation. Although transport is a potential source of physical and psychological stress for all horses, the risks of mortality, disease, and injury are generally higher for low-value animals, such as meat horses, which are often transported in unsatisfactory conditions [4,5]. Moreover, several countries have no plants that slaughter horses for human consumption. As a result, each year, hundreds of thousands of horses are subjected to a gruelling, cross-border journey (>8 h of travel) that ends in slaughter [2,3,6,7,8]. Long journeys increase the risk of welfare issues and often lead to blurring of information related to transport conditions [8].

The effects of transportation on the welfare of horses include anxiety-related behaviours, aggression, exhaustion, injury, respiratory and gastrointestinal disease, dehydration, pyrexia, and immunosuppression [3,9,10]. About 1% of horses die en route [4] but a greater percentage of animals are euthanised later due to severe injuries sustained during the journey or have non-visible injuries such as bruising, which is only recognisable post-mortem [11]. Behavioural and physiological responses, as well as injury rates, are affected by management factors such as vehicle specification, journey duration, and driver experience [12,13,14], and also by the physical fitness of the horse, its temperament, and its coping strategies [3,5,10,15,16]. For example, Grandin et al. recommended that aggressive horses should be segregated during transport because fighting was documented as a major cause of injury [17]. Fazio et al. evaluated the stress responses of stallions with different temperaments and found that nervous stallions had poor capacity to adapt to transport, probably as a result of adrenocortical depletion [18]. Conversely, changes in physiological parameters and problem behaviours decrease with repeated transport, suggesting that transport-induced stress responses are reduced in horses habituated to the situation [19,20].

The influence of prior handling on the degree of transport stress experienced by horses has not been extensively studied. It is known, however, that abilities and coping strategies differ for broken (tamed) and unbroken (untamed) horses [21,22,23,24]. The response of unbroken horses to challenging situations is characterised by high arousal, fear, excitability, and a negative emotional state, increasing their risk of distress and travel-related pathologies. Knowles et al. [5] confirmed that transport management of unbroken ponies should take into account their ethology and physiological response to stressors. For example, unlike handled horses, it is preferable for untamed ponies to travel as a group [5]. It is likely that their strong herding instinct underlies this difference [5]. The same authors realised the importance of having a tool that would predict individual horse’s responses to transport stress before embarking on the journey. These authors failed to find a strong relationship between pre-transport behaviours and aggressive behaviour during transport. However, rating the reactions of untrained horses during a novel object or handling test may be a better predictor of their temperament, emotional responses, and coping strategies [23].

The European Union recognises that broken and unbroken horses have different needs and adaptability skills. For this reason, Regulation EC 1/2005 on the protection of animals during transport includes stricter rules for the transportation of unbroken animals: they must not be transported on journeys over eight hours, tied during transport, or transported in individual bays, but must instead travel in groups of ≤4. An unbroken horse is defined in the Regulation as a horse that “cannot be tied or led by a halter without causing avoidable excitement, pain or suffering”. However, this definition is not accompanied by verification procedures; therefore, in practice, there is still no test to identify whether a horse is broken or unbroken. Thus, even when violations are identified during on-road inspections, nobody can be fined because official veterinarians do not have a test to identify whether a horse is broken or not. The European Parliament has expressed serious concerns about horse welfare during transportation and admits that there is still a high level of regulatory noncompliance, mainly related to unbroken horses [2,4]. It is therefore essential to provide official veterinarians with a tool that allows them to categorise horses and, consequently, direct transporters towards the correct transport procedures. Provision of a reliable test and the resultant reduction in the number of horses that travel under inappropriate conditions would avoid many injuries and substantial suffering.

The objective of this study was to develop and validate a behavioural test to identify whether a horse is broken or unbroken. The study was based on the hypothesis that horses show different behavioural and physiological responses to being approached, haltered, and led, depending on prior level of tameness.

## 2. Materials and Methods

### 2.1. Sample Size Calculation

The sample size calculation was based on intraclass correlation coefficient (ICC) procedures, as previously reported [25]. The sample size was calculated using the confidence interval (CI) with the desired precision approach proposed by Bonnet [26] with a 95% CI of width Wk = 0.15, assuming a planning value of ICC (ICCplan) = 0.7 and k = 4 raters and an expected drop-out rate of 3–5%. The minimum sample size required was 100 horses.

### 2.2. Farms and Animals

Experiments were carried out during March and April 2021 on nine farms in Italy, with an average temperature and humidity of 18 °C (range: 9–26 °C) and 55% (range: 30–68%), respectively. The altitude of the farms ranged from 4 masl to 522 masl. The total number of horses reared on each farm ranged from 3–25 (mean, 11) and the paddocks where the horses were kept ranged from 15–10,000 m^2^. Only 11 horses were kept in individual housing (usually stallions) while the majority (78%) were kept in groups of ≥3 (maximum group size, 21). More than 50% of the horses had >133 m^2^ of space allowance (Table S1). In two paddocks, a stallion was kept with mares. Ten horses were confined in indoor stalls (stallions or mares before or after foaling) while the others were housed in outdoor paddocks with shelter. No horse was tied. Water and hay were provided ad libitum on all farms.

Italian Heavy Draft horses (n = 103) were selected for this study. Demographic data (i.e., age and sex) of the horses were provided by the owner (Table S2). Horses were born on the farm where they were assessed or had been kept there for at least three months prior to assessment. They were reared for various purposes (breeding, meat production, family enjoyment, and showing). Three horses were excluded from the study before the start of the test due to lameness. Thus, the final group consisted of 100 healthy horses (16 males and 84 females, including 14 broodmares with foals), without behavioural problems (i.e., aggressivity and stereotypy), with a mean age of 7 years (range 1–25 years). A subset of these horses (n = 65; 6 males, 59 females; age (mean/range), 7 years/1–25 years) was evaluated twice, with a 3-week interval between evaluations. Before the start of the testing procedures, one author (LM) asked each owner whether their horses were broken or unbroken, based on how much handling and training they had undergone. According to the owners’ opinions, 55 of the 100 horses (55%) were broken (6 males, 49 females; age (mean/range), 8 years/1–25 years) and 45 (45%) were unbroken (10 males, 35 females; age (mean/range), 5 years/1–18 years).

### 2.3. Testing Procedure

Each horse underwent a Broken/Unbroken Test (BUT), which consisted of two phases (Approaching and Haltering Test (AHT); Handling Test (HT)) to evaluate their behaviour during approaching, haltering, and leading. The BUT was always performed by the same tester (BP), a veterinarian with more than 20 years of experience in horse behaviour, handling, and learning theory, and who was trained for the procedure. The tester was unfamiliar to the horses and blinded to their owner-assessed level of handling. Each horse was tested in the paddock/pen where it was usually kept (test area). Horses were usually kept without a halter on. For biosecurity reasons, halters were provided by each farmer. Thus, two different types of halter were used; all were adjustable at the headpiece, but 17 had a clip at the throatlash and 83 did not.

**Figure 1:**
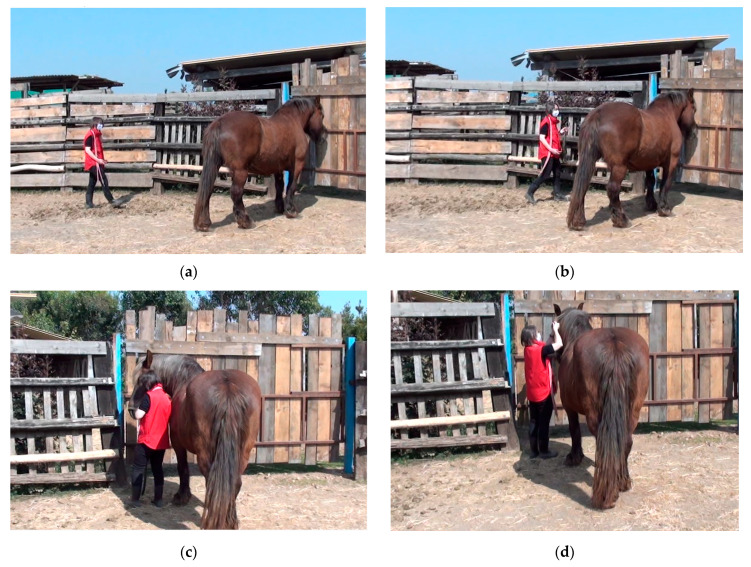

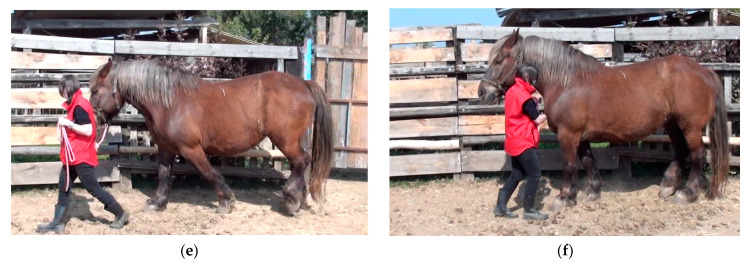
The Broken/Unbroken Test (BUT) consists of two phases: in the first phase (**a**–**d**, Approaching and Haltering), the tester entered the test area, walked towards the horse slowly with the halter in her hand, approached the horse, and then tried to halter it; in the second phase (**e**,**f**, Handling), which was only attempted if it had been possible to complete the first phase within 5 min, the tester tried to lead the horse three steps forwards and three steps backwards. In a real setting, the tester should wear protective equipment.

The BUT was a modified version of the tolerance test proposed by Wulf et al. [27]. Briefly, the tester entered the test area and walked towards the horse slowly with the halter in her hand, approaching and then trying to halter the horse (AHT; Figure 1a–d). The maximum time allowed for approaching and haltering the horse was 5 min. If it was not possible to approach and halter the horse within this time, the test ended. If it was possible to halter the horse within the maximum time, the tester started the second phase of the test (HT). During HT, the tester tried to lead the horse three steps forward (about 2 m distance) and three steps backwards (Figure 1e,f). When leading, the handler kept the horse relatively close to her right-hand side. The tester used a negative reinforcement procedure, applying light pressure on the lead rope and releasing the pressure as soon as the horse showed the desired behaviour (e.g., a step forward). The maximum time allowed for HT was 5 min. Thus, the maximum total time for BUT was 10 min. However, the procedure was stopped at the first sign of pain or if the horse showed a high level of distress (shaking movements of the head and tail, rearing, or moving away vigorously) or signs of aggression (ears laid backwards, attempting to bite or kick). A collaborator with experience in horse behaviour and handling used a stopwatch (Onstart 310, Kalenji, Decathlon, Villeneuve d’Ascq cedex, France) to record the time required for completion of each phase of the test and alerted the tester if the maximum time had elapsed or if the horse was displaying the aforementioned behaviour consistent with pain, distress, or aggression. All tests were filmed using a digital video camera recorder (HDR-CX115E, Sony, China) for later analysis. To evaluate test–retest reliability, the BUT was repeated 3 weeks later, by the same tester and under the same experimental conditions, on a subset of the original group of animals.

At the end of the BUT, the tester judged whether the horse was broken or unbroken based on her own experience and knowledge. This ‘expert judgment’ was based on the horse’s ability to respond to pressure, allowing the handler (tester/expert) to control the horse’s head and front and back legs in accordance with learning theory [28]. The expert’s judgment as to whether the horse was broken or unbroken was treated as the ‘gold standard’ in the analyses.

### 2.4. Observers and Training

All BUT videos were renamed and coded using casual numbers by an author (LM) not involved in scoring, and then analysed by four condition-blind (e.g., broken or unbroken, ID of horses and farmer) observers. One observer (main observer, EDC) was a senior veterinarian with long experience in evaluating horse welfare and behaviour, while the last three were veterinary or animal science students (non-expert observers). The latter were all familiar with horses but had no experience in formal behavioural observations. The non-expert observers were trained by the main observer on the behavioural scores to apply (Table 1) using 30 videos. The observers were trained on the fact that if a phase of the test (AHT or HT) was not completed within the maximum time or was stopped due to pain and/or distress, a score of 0 (worst situation) must be attributed to that phase. Moreover, when AHT received 0, HT must receive 0 as well. The training ended after approximately 15 days when an excellent level of inter-observer agreement for each score (kappa > 0.75%; [29]) was obtained.

### 2.5. Test Scoring and Classification of Horses According to Regulation EC 1/2005

Each of the four observers assessed each of the AHT and HT videos recorded during the data collection phase of this study, using the criteria in Table 1. These assessments were made independently. The behavioural score for each phase of the BUT was based on a three-point scale (0–2). The AHT and HT scores were then summed to obtain an overall BUT score (0–4).

After watching the videos, the observers also classified the horses as broken or unbroken according to the definition provided by Regulation EC 1/2005: “An unbroken horse is a horse that cannot be tied or led by a halter without causing avoidable excitement, pain or suffering” [30].

The observers scored 100 videos showing the first BUT and 65 videos showing the repeated BUTs on a subset of the same horses, to evaluate test–retest reliability. Of these 165 videos, 20 were randomly selected, renamed, and analysed for a second time to estimate intra-observer agreement. Therefore, overall, the observers scored a total of 185 videos.

### 2.6. Physiological and Behavioural Parameters for Validation

Data relating to several physiological and behavioural parameters were collected during the BUT procedures for validation of the behavioural scores. During the approach phase of the AHT, the distance between tester and horse, at the moment when the horse showed any avoidance behaviour (e.g., moving away, turning the head away), was measured, as reported by Dalla Costa [31], using a laser distance meter (LV5800-50M, LOMVUM, China). This was defined as the ‘avoidance distance’ (Figure 1a,b). If the tester touched the horse, the avoidance distance was considered as 0 m. When possible, the tester also evaluated the horse’s heart rate (HR) (facial artery) and respiratory rate (RR) (flank movement) [32] at the end of the BUT. These parameters required touching the animal and could not be evaluated in all horses. In particular, HR data were missing for most of the horses, which were judged by the expert as unbroken (41/49 and 25/26 of horses during the first and second tests, respectively).

**Figure 2:**
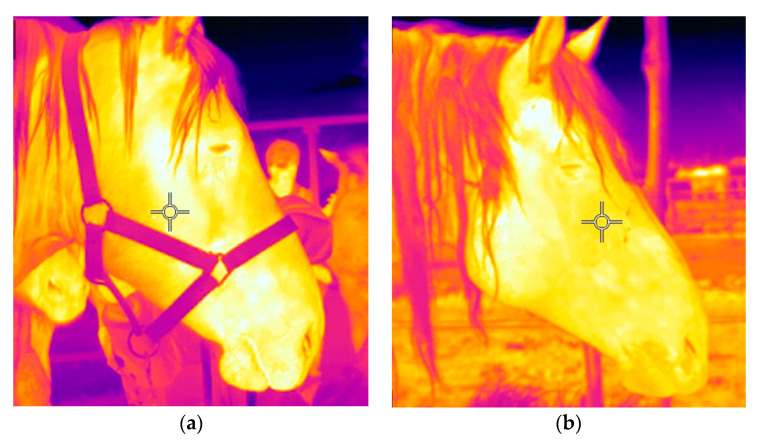
Infrared thermography images collected at the end of Broken/Unbroken Test (BUT) in haltered (**a**) and non-haltered horses (**b**).

When possible, three infrared thermography images of the horse’s head were taken at the end of the BUT, by the same person (LM), using a portable camera (FLIR E76 24°; FLIR Systems AB, Danderyd, Sweden), from the right side of the horse (Figure 2). The camera was calibrated using the environmental temperature and relative humidity recorded on that day by a weather station (Kestrel 4000, USA). Illuminance was recorded using a lux meter (BTMETER 881E, Aquarium Photography, China). The resolution of the camera was 320 × 240 pixels, and the accuracy was ±2°C or ±2% at environmental temperatures ranging from 15 to 35 °C. The camera was positioned at 90° to the sagittal plane and, in order not to scare the less well-handled horses, at a distance of approximately 3 m from the horse’s side. The exact distance from the horse was recorded using the camera’s distance laser meter. The images were analysed using the FLIR Tools^®^ software (FLIR Systems, Inc., Täby, Sweden). In particular, an oval area was traced around the eye, including the eyeball and ∼1 cm around the outside of the eyelid, and the maximum temperature (°C) in this region was recorded, as previously reported [33,34,35]. Eye Temperature (ET) was defined as the mean of the three maximum temperatures (one per image). Similar to HR values, ET could not be calculated for all horses because many of them drifted away during the test and could not be re-approached within 3 m. This parameter was calculated for 91/100 and 60/65 horses for the first and second tests, respectively.

In addition to being scored by each of the four observers, the BUT videos were assessed by one author (LM) for the following timings using a stopwatch (Onstart 310, Kalenji, Decathlon, Villeneuve d’Ascq cedex, France):

- Approach time: This was defined as the time taken for the tester to move from a distance of about 3 m from the horse to the point that she was able to gently touch the horse’s shoulder. Usually, the tester gently and slowly raised her hand to signal the start of the procedure (and therefore the start of the approach time).
- Haltering time: This was defined as the time that elapsed between the end of the approach time (i.e., the tester touching the horse’s shoulder) and the halter being correctly positioned. This latter moment was generally apparent in the recording as the sound generated by clipping the halter, or by the tester saying ‘OK’ to signal the end of the haltering procedure.
- Handling time: This was defined as the time required for the tester to lead the horse forwards and backwards. The time was measured from the end of haltering (i.e., correct positioning of the halter) to the end of the handling test (i.e., the horse having completed three steps forwards and three steps backwards). Usually, the tester saying ‘OK’ to signal the end of the test.
- Total time: This was calculated by summing the approach, haltering, and handling times.

If, during the AHT or HT, the horse showed pain and/or a high level of distress (e.g., moving away vigorously, intending to kick), the test was terminated, and the maximum time (5 min) was automatically assigned to the test.

### 2.7. Analytic Approach

Statistical analyses were performed with SPSS Statistics version 25 (IBM, SPSS Inc., Chicago, IL, USA). The level for statistical significance was set at *p* < 0.05. Statistical methods for validation of the BUT scale are summarised in Table 2, in agreement with the literature [36,37,38].

Initially, descriptive statistics were performed, and values were expressed as numbers and percentages and as mean ± standard error.

Validation of the BUT score started with the assessment of inter-observer, intra-observer, and test–retest reliability, as well as internal consistency [36]. Inter-observer reliability for the AHT and HT scores was estimated using the Fleiss’ kappa (*F_k_*) [40], as this test allows evaluation of the agreement between >2 observers and provides an estimate of the agreement for each score (0, 1, and 2) of the scale. The 95% CI for *F_k_* was also calculated. *F_k,_* was also used to evaluate inter-observer agreement for horse classification (broken/unbroken) based on the definition written in the EU legislation. The BUT score was treated as a continuous variable and analysed using the consistency type ICC using a two-way mixed model approach [38]. Values < 0.4 indicate poor reliability, those between 0.40 and 0.75 represent fair-to-good reliability, and values > 0.75 indicate excellent reliability [41].

Intra-observer and test–retest reliability for the AHT and HT scores and the classification based on the Regulation’s definition were evaluated using concordance rate and Kendall tau-b correlation coefficient (τ), a measure of association based on the number of concordances and discordances in paired observations. The τ could range from 0 (no concordance) to 1 (perfect concordance). Associations were considered weak if τ < 0.30, moderate if 0.30 ≤ τ ≤ 0.50, and strong if τ > 0.50 [42]. The ICC using the two-way mixed model approach was used to evaluate intra-observer and test–retest reliability for the BUT score [43].

Internal consistency was estimated by the inter-test correlation (i.e., inter-item correlation) evaluated using Spearman’s rank-order coefficient (ρ) as Cronbach’s coefficient alpha is inappropriate and meaningless for two-item scales [39]. Correlations of each item and BUT score were also calculated (i.e., item-BUT score correlation). On a reliable scale, each item should have a ρ > 0.30 [44].

The second phase of validation focused on validity. Validity was evaluated by using the BUT score of the main observer while the expert’s judgment (broken/unbroken) was treated as a criterion measure.

Construct validity was determined using the hypothesis that there would be a negative correlation between BUT score and ET, HR, RR, avoidance distance, as well as approach, haltering, and handling times. In other words, we expected that these parameters would decrease as the level of previous tameness increased, and, consequently, as BUT score increased. These associations were evaluated using Spearman’s coefficient (ρ). This was only performed using data from the first test session, to avoid possible bias due to repeated measures. The correlation was considered poor if ρ < |0.3|, medium if |0.3| ≤ ρ < |0.5|, and large if ρ ≥ |0.5| [44]. The association between BUT score and physiological and behavioural parameters was also analysed using ordinal logistic regression. Generalised Linear Models (GLMs) procedures with a multinomial probability distribution and cumulative logit link function were used, with BUT score as the dependent variable and physiological and behavioural parameters as predictors. Moreover, since ET could be affected by many intrinsic and exogenous factors, especially when measured under field conditions [34], this was further investigated using GLMs with a normal probability distribution and identity as link function. ET was included as a dependent variable while the following multiple fixed effects were evaluated: BUT score, age, sex, exact distance between camera operator and horse, environmental temperature, and lux. In these GLMs, the horse was included as the subject and the session (first or second) as the within-subject effect. Results were expressed as odds ratio (OR), 95% CI, and the P-value of the Wald statistic.

Criterion validity indicates the accuracy in predicting scores on a ‘gold standard’ criterion measure (expert’s judgment) [36]. The sensitivity of the BUT score as a predictor of horses’ tameness level was evaluated by binary logistic regression, using GLM procedures, and expressed as OR, 95% CI, and P-value. The hypothesis was that the BUT score would increase in line with the tameness level of the horse (i.e., the odds that the horse was broken would increase as the BUT score increased). The expert’s judgment (broken/unbroken) was included as the dependent variable and the main observer’s BUT score as the predictor. The horse was also included in the model as the subject and the session (first or second) as the within-subject effect.

Receiver operating characteristic (ROC) analysis was used to estimate the ability of the BUT score to discriminate between broken and unbroken horses and to determine the optimal threshold value (cut-off) for discriminating between the two groups. The condition of ‘broken’, as defined by the expert, was set as the positive actual state and larger values of the BUT score indicated stronger evidence for a positive actual state. Based on the area under the curve (AUC), the BUT score may be considered to be uninformative (AUC = 0.50), poorly accurate (0.50 ≤ AUC ≤ 0.70), moderately accurate (0.70 ≤ AUC ≤ 0.90), very accurate (0.90 ≤ AUC < 1) or perfect (AUC = 1). The optimal cut-off was determined using Youden’s index [45,46].

Finally, we evaluated agreement in horse classification (broken/unbroken) between the expert’s judgment, the owner’s opinion, the classification according to the Regulation as assessed by the observers after viewing the videos, and the BUT score according to its optimal cut-off. In order to define the agreement between each of these classifications, a correlation matrix was built reporting the Cohen’s kappa (*C_k_*) values for each pair of comparisons [47]. Only the first session was included in this analysis. As for the reliability analyses, values below 0.4 were considered as poor agreement, values above 0.75 as excellent agreement, and values between 0.4 and 0.75 as fair-to-good agreement [41].

## 3. Results

### 3.1. Descriptive Statistics

**Figure 3:**
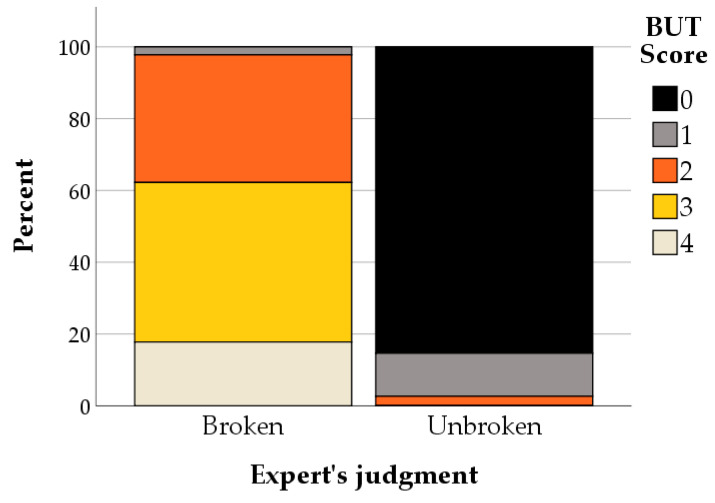
Relative distribution of the main observer’s BUT score according to the expert’s judgment of broken vs. unbroken status.

Descriptive statistics of AHT and HT scores, BUT scores, and classification of horses according to the Regulation EC 1/2005 definition by the four blinded observers are shown in Table S3. Figure 3 shows the relative distribution of the main observer’s BUT score for horses judged by the expert as being broken vs. unbroken. None of the horses classified as broken had a BUT score of 0, while > 40% had a BUT score of 3. Among the horses classified as unbroken, none had a BUT score ≥ 3, while 85.3% had a BUT score of 0.

### 3.2. Reliability of Test Scores, BUT Score, and Classification of Horses According to Regulation EC 1/2005

Table 3 shows the results of the inter-observer agreement analyses for the AHT and HT scores. Inter-observer reliability of test scores ranged from 0.652 (score 1 of AHT) to 0.961 (score 0 of HT). The overall inter-observer reliability was, however, excellent for both tests (*F_k_* > 0.750, *p* < 0.001).

Table 4 shows indices for intra-observer and test–retest reliability for the AHT and HT. Concordance rates for intra-observer reliability exceeded 90%, and correlations were very high (τ > 0.8) for both tests. Concordance between the first and second sessions was approximately 75%, but all concordances were ‘strong’ according to the τ values (τ > 0.5).

Table 5 presents the reliability indices for the BUT score. ICC ranged from 0.792 for test–retest agreement to 0.916 for inter-observer agreement, with all indices indicating excellent reliability (ICC > 0.750).

A strong correlation was found between the two AHT and HT scores (ρ = 0.825; *p* < 0.01) as well as between each of these test scores and the BUT score (Approaching and Haltering-BUT: ρ = 0.948; Handling-BUT: ρ = 0.951; *p* < 0.01). These findings support the internal consistency and the homogeneity of the BUT score.

Finally, the reliability indices for classification of broken/unbroken according to the Regulation showed poor inter-observer agreement (*F_k_* = 0.536, 95%CI = 0.477–0.596; *p* < 0.001), excellent intra-observer agreement (concordance rate = 93.8%; τ = 0.864; *p* < 0.001), and good test–retest reliability (concordance rate = 79.6%; τ = 0.592; *p* < 0.001).

### 3.3. Validity of Total Score

Descriptive statistics of physiological and behavioural parameters are shown in Table 6, while Table 7 shows their correlations with each other and with BUT score. BUT score was negatively correlated with most of the physiological and behavioural parameters investigated. In particular, BUT score increased as RR, avoidance distance, and all recorded times reduced (for all; *p* < 0.01). In contrast, no correlation between BUT score and HR or ET was found. However, GLM revealed that ET was influenced by exogenous factors: it increased as environmental temperature increased (OR = 1.096, 95%CI = 1.025–1.099; *p* = 0.001) and as distance from the horse decreased (OR = 0.632, 95%CI = 0.521–0.766; *p* < 0.001; Table S4).

**Figure 4:**
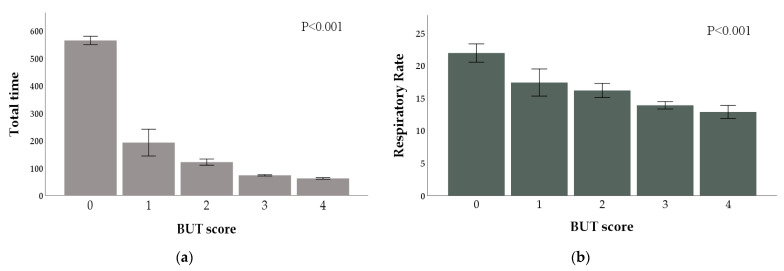
Means and standard errors of Total time (sec) (**a**) and Respiratory rate (bpm) (**b**) by BUT score. Ordinal logistic regression showed that, as Total time and Respiratory rate decrease, the probability of a higher BUT score increases (*p* < 0.001).

Ordinal logistic regression also showed that reductions in RR and all the recorded times were associated with an increase in the odds of higher BUT scores (OR < 1.0; *p* < 0.05; Figure 4 and Table S5). The narrow confidence intervals of these ORs indicate a high level of precision of the estimated effect.

Criterion validity was evaluated using binary logistic regression to determine the nature of the relationship between BUT score and the ‘gold standard’ criterion. This analysis showed that, for every one-point increase in BUT score, the likelihood that the horse had been classified by the expert as broken increased more than 90-fold (OR = 94.719, 95% CI = 19.748–454.308; *p* < 0.001).

**Figure 5:**
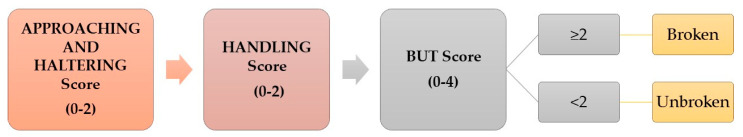
Proposed procedure to determine whether a horse is broken or unbroken. The horse’s behaviour during the Approaching and Haltering and Handling Tests should be scored on a 0–2 scale. The two scores are then summed to obtain the BUT score (range, 0–4). If the BUT score is ≥2, the horse should be identified as broken; if the BUT score is < 2, the horse should be identified as unbroken.

Moreover, ROC analysis showed that the BUT score was a very accurate method to discriminate between broken and unbroken horses (AUC = 0.993, 95%CI = 0.984–1.000; *p* < 0.001; Figure S1). The optimal BUT score cut-off value to discriminate between the two groups was 2 (i.e., ≥2 indicates ‘unbroken‘, <2 indicates ‘broken’). This cut-off has a sensitivity of 97.8% and a specificity of 97.3%. Figure 5 summarises the procedure for assessing whether a horse could be identified as broken or unbroken according to the optimal cut-off.

The agreement between horse classification based on the optimal BUT score cut-off (broken if BUT score ≥ 2) and expert judgment is shown in Table 8. Based on this cut-off, 50/100 horses were classified as unbroken, and the classification of only 3 horses was not in agreement with the expert’s judgement. The BUT score showed, thus, the highest degree of accuracy. Contrariwise, the lowest agreements were found for the Regulation definition.

## 4. Discussion

This study describes the development and validation of a test (BUT) that—for the first time—allows the classification of horses as broken or unbroken. Our results confirmed our hypothesis that horses with different levels of prior handling would react differently to being approached, haltered, and handled. The BUT is based on scoring the horse’s behaviour when it is approached, haltered, and handled in a standardised way. Each horse receives a score that ranges from 0 to 4, where 0 indicates the worst situation while 4 indicates the best situation. Thus, low BUT scores were assigned to horses that showed nervousness and avoidance behaviours, as well as those that could not be approached, haltered, and led, whereas high BUT scores were assigned to horses that exhibited fewer avoidance behaviours and could be approached, haltered, and led easily. We established a threshold that allows classification of a horse as broken (BUT score ≥ 2) or unbroken (BUT score < 2). This simple test could fill a legislative gap as, although Regulation EC 1/2005 includes different rules for transport of broken and unbroken horses, no tool for the classification of horses has previously been available. Due to their greater reactivity, unbroken animals are at increased risk of injury and disease. However, if the BUT was included in future transportation regulations worldwide, it would ensure that the correct transport procedures were followed for these animals and would help officials to verify regulatory compliance. Regular application of the BUT before a journey to a sub-group (randomly selected) of the horses in departure could therefore safeguard the horses’ welfare.

The current study followed the rigorous validation process that is required to confirm the reliability and validity of behavioural rating scales [36,37,38]. Agreement analyses showed that the AHT, the HT, and the BUT all have excellent inter-observer and intra-observer reliability. The highest agreement indices were obtained for score 0, while the lowest were obtained for score 1. These findings are not surprising because a score of 0 indicates not only high arousal levels (e.g., aggressive responses, fear, excitation) but also test failure (animal not haltered or led), a situation that is well-defined and unambiguous. Conversely, a score of 1 indicates an intermediate arousal level (moderate reluctance and one or more avoidance behaviours) where subjective judgment could have a greater influence. This is the first test developed to assess the horses’ level of prior tameness, and there is no literature with which to compare the results directly. However, similar values of inter-observer agreement have been reported for tests evaluating the human-animal relationship [31] and for some pain scales [25,48]. Czycholl et al. [49] have recently evaluated inter-observer reliability of the indicators proposed by the AWIN protocol for horses, including behavioural tests scored on a 3- or 2-point scale. The authors reported acceptable-to-good agreement for all the indicators but highlighted that behavioural responses such as fear and avoidance, as well as approach tests, may show low reliability in horses because—similar to other species [50,51]—they may show incongruent behaviours (e.g., the simultaneous existence of curiosity and fear could lead them to approach, then run away, and then return). However, although defining an exact level of arousal may be problematic, our experience shows that rating horses’ behavioural responses with the BUT is a reliable and easy way to judge the level of prior handling and tameness of unfamiliar horses.

Test–retest reliability data were obtained by repeating the BUT on a subset of horses three weeks after the first session. Although test–retest reliability was lower than inter- and intra-observer reliability, it was good for the AHT and the HT, and excellent for the BUT. This result was expected as it is commonly accepted that test–retest could be influenced by many factors including changes in test conditions, physical or mental state of the animal, and its learning experience [36,52,53]. In the present study, the main sources of variability affecting test–retest agreement were likely to be intra-observer variation and changes in the horses’ behavioural responses. Even in a well-defined test situation, test–retest reliability is very sensitive to the animal’s affective state and mood on the day. For example, a positive emotional state could imply a lower latency time in approaching humans and less aggressive and fearful behaviours [52,54], leading to a higher BUT score. Conversely, a negative emotional state leads to fearful and cautious reactions [52], which could result in a lower BUT score. In the repeated BUTs, although many days had passed between the two tests, a positive or negative emotional response could also be linked to the horse’s memory of its previous BUT experience [50,53]. Pain is another factor that could influence the horse’s response to human approach [31,55]. To limit this bias, only horses that appeared to be healthy on visual clinical evaluation were used. Moreover, although tester and test area were the same in the two repetitions and no major changes had occurred within the farms, identical test conditions could not be guaranteed. For example, changes in social dynamics of the herd or the farmer’s handling between the two sessions could have affected the horses’ behaviour and, therefore, the test scores. Most horses were tested in the presence of other animals, and this could also confound the results. Unfortunately, none of these factors could be controlled in the present study, and this may explain the relatively low test–retest reliability. In spite of these caveats, the agreement indices indicate that the horses’ responses to the BUT were consistent over time. We suggest, however, that assessors in the field should take into account the environmental and psychological context in which the test is conducted during their scoring, as the stability of the BUT across different situations has not been confirmed. We also suggest that the tester wear protective equipment and always stop the test at the first signs of distress or aggressive behaviour.

Our BUT demonstrated both construct and criterion validity. The correlation between BUT and the recorded physiological and behavioural measures, which are known to be related to the level of taming [21,22,23], confirmed its construct validity [36,37]. Some authors have indeed claimed that the different reactions to stimuli of “naïve” horses compared to trained ones are related to activation of different areas of the brain [22]. In particular, broken horses do not usually show negative reactions when exposed to humans or novel environments, whereas unbroken horses typically show a high level of emotion and display different behavioural (aggression, fear, vocalisation, defaecation, and so on) and physiological (changes in heart rate, blood pressure, hormones, respiration rate, and so on) stress related responses [21,24,56,57]. Several authors [23,58,59] have shown that, compared with unbroken horses, broken horses have a lower increase in heart rate, approach the tester sooner, and are caught more quickly than unbroken horses during novel object and handling tests. It has also been shown that a reduction in emotional reactivity and improvement in the human–horse relationship continue as the number of training and handling sessions increases [60,61]. Our correlation analyses have confirmed that a high BUT score indicates a broken horse, as this was associated with lower respiratory rates, avoidance distances, as well as the time taken for approach, haltering and handling. Conversely, horses achieving a low BUT score could be defined as unbroken because they showed high RR, avoidance distance, and test times. However, there was no correlation between BUT score and HR or eye temperature. HR has been used previously to assess the personality and reactivity of horses [23,60], including those that are unbroken [58,59]. The inconsistency between our results and those of previous studies is likely to be due to the amount of missing HR data in our study. Its measurement required close contact with the animal and therefore could not be collected for many horses, particularly those that were unbroken, as the test was stopped if the horse showed signs of distress (e.g., flight response). Moreover, HR in horses increases during physical exercise, and, after the BUT, it could have been difficult to distinguish between emotional and physical reasons for an increase in HR. Eye temperature, on the other hand, has been used as an indicator of arousal for horses [33,35,60], but this can be influenced by many factors, especially when measured in the field [34]. We tried to standardise some of these factors; however, this was not always possible under field conditions. Indeed, ET was still strongly influenced by environmental temperature and measurement distance, and these could represent confounding factors that mask its association with the animal’s level of reactivity. In the context of the present study, parameters that do not require close contact with the animal seem not only feasible but also fit for purpose. Higher respiratory rates, the presence of avoidance behaviour, and longer approach times for unbroken horses indicate activation of the sympathetic nervous system and a “fight-or-flight” response. They also suggest that these horses perceive approach by a human as a dangerous situation that triggers a negative emotional state and a high degree of arousal [24]. Fearful and stressed horses are more likely to develop transport related respiratory disease after 8 h journey [62], so unbroken horses may be at higher risk when travelling over this lenght. The criterion validity was investigated by choosing the expert’s judgment as the ‘gold standard’ criterion measure against which to compare the BUT score. The expert evaluated the horse in the test area and defined it as broken or unbroken based on its ability to respond to pressure, used as negative reinforcement [28]. Our findings confirm the responsiveness and predictive value of the BUT on the basis that the likelihood of the horse being broken increased substantially with each additional BUT score point. The responsiveness of the BUT score also suggests that it could be used to indicate different levels of prior taming. For example, a BUT score of 0 could be defined as “no taming,” while scores of 2 and 4 could be defined as “moderate level” and “good level” of taming, respectively.

The utility of the BUT as a tool for widespread use is that it can discriminate, with high sensitivity and specificity, between a broken horse and an unbroken one. Specifically, a horse would be defined as broken if its BUT score was ≥2 and as unbroken if it was <2. Our statistical approach thus confirmed the criterion validity of the BUT and suggested a rigorous procedure for applying it. After adequate training, official veterinarians would be able to score a horse’s behaviour objectively using the BUT, decide whether the horse is broken or unbroken, and advise on the transport procedures that should be put in place. In addition to BUT’s binary classification (broken vs. unbroken), which should direct personnel towards using specific transport procedures, the BUT score may indicate the horse’s level of taming. This information could accompany the animal throughout its transport and could also be relevant to human safety, because the fearful and aggressive reactions that characterise a low level of taming have been identified as the major cause of horse-related accidents [21].

The results achieved so far proved that the BUT could be a reliable and valid tool. In contrast, when the observers were asked to classify horses using the definition proposed in Regulation EC 1/2005, all the agreement analyses showed that this classification system had poor reliability. It follows from this that the definition of unbroken horses, as written in the current legislation, is unclear. This could have led to confusion and consequently to the transport of unbroken horses over long distances and in inappropriate transport conditions [2,3]. This highlights the need to include, within the ongoing revision of the current legislation, a better definition of unbroken horses. However, we would also like to question the terminology that is currently used to define horses’ prior level of handling and training (i.e., taming). The term ‘unbroken’ was used in this study because it is the term used in Regulation EC 1/2005 to describe untamed horses. However, the converse state (‘broken’) implies that the animal has been ‘defeated’, ‘beaten’, ‘overpowered’, or ‘vanquished’, a terminology that is outdated at a time when humane animal handling and training procedures are prevailing worldwide. The term ‘broken’ is also used to indicate that the horse has been trained for riding or driving, something that is irrelevant in the context of this legislation. We, therefore, suggest that the term ‘unbroken’ should be replaced with ‘unhandled or untamed’ in the updated version of Regulation EC 1/2005.

Our findings need to be interpreted with caution because this study has several limitations. The BUT was applied to a draught horse breed, and all horses were tested in their paddock. Consequently, our findings need to be confirmed by applying the BUT on a larger population of horses housed in both familiar and unfamiliar environments. Untamed meat horses are often conducted in unfamiliar pens and kept there with a low space allowance before loading, so the BUT should be also re-conducted in a real setting; during the application of the BUT in a real-world, many other problems could happen, which may require a refinement of the described procedures. However, even if our results are preliminary, they confirmed that tamed and untamed horses have a different reaction when approached, haltered, and led. These differences in reactivity and their relationship with humans suggest that—as is currently the case under Regulation EC 1/2005—different transport procedures must be followed in these two groups of horses. This would help to reduce the distress that can be associated with the transport of horses with different levels of taming prior to transport. Since there is a need for a robust procedure that allows identification of these animals, based on our findings, it may be suggested to include the BUT in the legislation on the protection of welfare during live animal transport. This will allow personnel to define, prior to shipping, whether a horse is broken/tamed or unbroken/untamed, thus paralleling the current requirement for pre-transport assessment of fitness for travel. BUT test would take a bit of time during the preparation phase of transport; however, this little time investment may be crucial to safeguard the welfare of the travelling horses as well as the horse handlers, who often get injured during loading and unloading procedures. Horses and human health and welfare are indeed interconnected, and the application of BUT may therefore enhance both.

## 5. Conclusions

This study described the development of a reliable and valid behavioural test (BUT) that seems to be able to estimate a horse’s prior level of taming and classify it as broken or unbroken. The BUT was associated with excellent inter- and intra-observer agreement and test–retest reliability, and its construct and criterion validity are supported by its association with physiological and behavioural parameters and expert judgment. By contrast, our study suggests that the current definition of unbroken, as written in Regulation EC 1/2005, is not fit for purpose. Inclusion of the BUT in future codes for animal transportation and regular application of the BUT before shipping could help transporters to direct horses towards the correct transport procedures, help competent authorities to verify compliance with the Regulation, and—most importantly—allow horses to travel under conditions that are appropriate for their prior experience, thereby avoiding many injuries and much suffering. Ultimately, widespread adoption of the BUT could safeguard the welfare of millions of horses during transport and also minimise horse-related injuries to humans. However, further studies are needed to confirm our findings and to optimise transport strategies for unbroken horses and evaluate the effects of handling, loading, and travelling training on the horses’ emotional state and the incidence of transport-related problems.

## Figures and Tables

**Table 1:** Scoring system for classification of horses’ behaviour during the Approaching and Haltering Test (AHT) and Handling Test (HT), and calculation of the Broken/Unbroken Test (BUT) score.

Test	Score	Displayed Behaviour/Calculation Method
APPROACHING AND HALTERING (AHT)	0	The horse displays pronounced excitement or avoidance behaviour (shaking movements of the head, moves away or runs away vigorously, intends to kick) and cannot be approached or haltered successfully ^1^
	1	The horse displays moderate excitement and/or avoidance behaviour in response to approaching and haltering (one or more occurrences of licking, raising and shaking the head, moving away from the handler) but is haltered successfully
	2	The horse does not show any avoidance behaviour during approaching and haltering and is haltered successfully
HANDLING (HT)	0	The horse is not harnessed or displays pronounced reluctance and avoidance behaviours; the horse shows signs of aggression (ears laid backwards, tries to bite or kick), shows shaking movements of the head and tail, rears, moves away vigorously, or does not respond to the pressure correctly; the test is not completed successfully ^1^
	1	The horse displays moderate reluctance and avoidance behaviours in response to leading (moderate to strong pressure must be applied on the halter before the horse moves, horse raises the head, licks, moves away from the handler, swishes the tail at least once); the horse completes the test or part of it but with some difficulties
	2	The horse does not show any reluctance or avoidance behaviours, is led easily, and completes the test successfully
BUT SCORE	0–4	Sum of the AHT and HT scores

^1^ the test is stopped due to evident signs of distress or the time limit is reached before successful completion of the test.

**Table 2:** Statistical methods for validation of the Broken/Unbroken Test.

Reliability and Validity	Type of Analysis	Description	Statistical Test
-	Frequency distribution	Distribution of AHT, HT, and BUT scores	Descriptive statistics
Reliability	Inter-observer	Agreement among the four blinded observers ^1^	Fleiss’ kappa and ICC
	Intra-observer	Agreement between scores assigned by the same observer to videos viewed twice ^1^	Kendall tau-b correlation coefficient, concordance rate, and ICC
	Test–retest	Agreement between results of tests conducted on the same horse at two different times ^1^	
	Internal consistency and item-total correlation	Agreement between individual items of the scale ^1^ and between each item and the total score	Spearman’s rank-order coefficient ^2^
Validity	Construct	Degree to which the BUT score correlates with other measures to which it is theoretically related ^1,3^	Spearman’s rank-order coefficient and ordinal logistic regressions
	Criterion	Strength of the relationship between the BUT score and the ‘gold standard’ criterion ^4,5^	Binary logistic regression ^5^, Receiver operating characteristic (ROC) analysis, and Cohen’s kappa ^5^

AHT = Approaching and Haltering Test; BUT = Broken/Unbroken Test; CI = Confidence Interval; HT = Handling Test; ICC = intraclass correlation coefficient; ROC = receiver operating characteristic. BUT score = sum of scores assigned to AHT and HT tests. ^1^ Modified by Meagher [36]. ^2^ Spearman’s coefficient was chosen as Cronbach’s coefficient alpha is inappropriate for two-item scales [39]. ^3^ Convergent validity. ^4^ Modified by Boateng [37] (concurrent criterion validity). ^5^ Expert’s judgment used as criterion measure.

**Table 3:** Inter-observer reliability of scores for the four observers for the Approach and Haltering and Handling Tests (Fleiss’ kappa).

Reliability	Test	Category		Kappa	Lower 95% CI Bound	Upper 95% CI Bound	*p* Value
Inter-observer	Approaching and Haltering	Overall		0.794	0.752	0.837	<0.001
		Individual scores	0	0.955	0.897	1.014	<0.001
			1	0.652	0.593	0.711	<0.001
			2	0.733	0.674	0.792	<0.001
	Handling	Overall		0.756	0.712	0.799	<0.001
		Individual scores	0	0.961	0.869	0.986	<0.001
			1	0.790	0.615	0.733	<0.001
			2	0.662	0.531	0.648	<0.001

CI = confidence interval.

**Table 4:** Indices of intra-observer and test–retest reliability for the Approaching and Haltering and Handling Tests.

Reliability	Test	Concordance Rate	Kendall τ	*p* Value
Intra-observer	Approaching and Haltering	92.6%	0.877	<0.001
	Handling	92.5%	0.850	<0.001
	Overall	92.6%	0.866	<0.001
Test–retest	Approaching and Haltering	73.1%	0.703	<0.001
	Handling	75.4%	0.719	<0.001
	Overall	74.2%	0.717	<0.001

CI = confidence interval.

**Table 5:** Reliability indices of the BUT score.

Reliability	Intraclass Correlation Coefficient	Lower 95% CI Bound	Upper 95% CI Bound	*p* Value
Inter-observer	0.916	0.896	0.933	<0.001
Intra-observer	0.866	0.799	0.912	<0.001
Test–retest	0.792	0.741	0.833	<0.001

CI = confidence interval.

**Table 6:** Descriptive statistics for physiological and behavioural parameters.

Parameter	Expert’s Judgment		Overall
	Broken	Unbroken	
HR (beats/minute)	38 ± 1	45 ± 2	39 ± 1
RR (breaths/minute)	15 ± 1	21 ± 1	17 ± 1
ET (°C)	35.43 ± 0.08	35.23 ± 0.13	35.35 ± 0.07
Avoidance distance (m)	0.00 ± 0.00	0.44 ± 0.10	0.20 ± 0.05
Approach time (s)	14 ± 3	48 ± 8	29 ± 4
Haltering time (s)	42 ± 4	169 ± 12	100 ± 8
Handling time (s)	34 ± 1	258 ± 11	136 ± 10
Total time (s)	90 ± 5	510 ± 21	281 ± 19

ET = eye temperature, HR = heart rate, RR = respiratory rate. Values represent mean ± standard error.

**Table 7:** Spearman correlations (ρ) among BUT score, physiological parameters, and behavioural parameters.

	HR	RR	ET	Avoidance Distance	Approaching Time	Haltering Time	Handling Time	Total Time
BUT Score	−0.189	−**0.685** **	0.132	−**0.384** **	**−0.378** **	**−0.710** **	**−0.910** *	**−0.910** **
HR		**0.346** *	−0.034	NA	−0.016	0.060	0.263	0.136
RR			−0.133	0.189	0.201	**0.513** **	**0.528** **	**0.616** **
ET				−0.087	**−0.221** *	−0.141	−0.079	−0.119
Avoidance distance					**0.525** **	**0.408** **	**0.374** **	**0.388** **
Approach time						**0.343** **	**0.361** **	**0.487** **
Haltering time							**0.668** **	**0.793** **
Handling time								**0.918** **

HR = heart rate, RR = respiratory rate, ET = eye temperature. NA = not applicable as HR was only assessed when avoidance distance was 0. Bold values denote statistical significance at the *p* < 0.05 level: ** Correlation is significant at the 0.01 level (2-tailed), * Correlation is significant at the 0.05 level (2-tailed).

**Table 8:** Agreement in horse classification (broken or unbroken) among the judgment of the expert, the optimal cut-off of the BUT score, the classification according to the Regulation EC 1/2005, and the owner’s opinion (Cohen’s kappa).

	BUT Score ^1^	Owner’s Opinion	Regulation Definition
Expert’s judgment	0.940 ***	0.840 ***	0.662 ***
BUT Score ^1^		0.780 ***	0.680 ***
Owner’s opinion			0.554 ***

^1^ Classification based on optimal cut-off (broken if BUT score ≥ 2) *** *p* < 0.001.

## Data Availability

The data presented in this study are available in the article and supplementary material. Further information is available upon request from the corresponding author.

## Supplementary Materials

The following are available online at https://www.mdpi.com/article/10.3390/ani11082303/s1, Figure S1: Receiver Operating Characteristic (ROC) curve of BUT score for predicting broken vs. unbroken status in horses. Table S1: Housing conditions of the examined horses; Table S2: Demographic characteristics of examined horses b farms; Table S3: Descriptive statistics for AHT, HT, and BUT scores and classification of horses, by the blinded observers, according to the definition of ‘unbroken’ in Regulation EC 1/2005; Table S4: Factors affecting eye temperature (ET) of horses from the multivariable model including ET as dependent variable; Table S5: Ordinal logistic regression: factors associated with BUT score.
