# Supplementary material for: Development and Validation of a Test for the Classification of Horses as Broken or Unbroken
Source: Animals (Basel). 2021 Aug 4;11(8):2303. doi: 10.3390/ani11082303 (PMC8388372; doi:10.3390/ani11082303)
Supplement: Supplementary file 1 [file animals-11-02303-s001.zip › animals-1319510-supplementary.pdf]

Table S1. Housing conditions of the examined horses.

Parameter	Mean	Standard Deviation	Median	Minimum	Maximum
N° horses at the farm	11	7	10	3	25
Size of paddock (m^2)	1774.3	2032.5	1600.0	15.0	10000.0
N° horses in the paddock	8	7	4	1	21
Space allowance (m^2/animal)	475.0	1162.5	133.3	10.0	10000.0

Table S2. Demographic characteristics of examined horses by farms

Farm	Sex				Age (years)	
	Male		Female			
	Count	%	Count	%	Mean	Standard Deviation
1	1	5.0%	19	95.0%	7	4
2	1	10.0%	9	90.0%	7	6
3	1	20.0%	4	80.0%	8	7
4	0	0.0%	9	100.0%	5	3
5	7	63.6%	4	36.4%	3	3
6	0	0.0%	3	100.0%	5	4
7	1	20.0%	4	80.0%	9	5
8	0	0.0%	12	100.0%	10	9
9	5	20.0%	20	80.0%	6	6

Table S3. Descriptive statistics for AHT, HT, and BUT scores and classification of horses, by the blinded observers, according to the definition of 'unbroken' in Regulation EC 1/2005.

Parameter	Score/ classification	Observer							
		1		2		3		4	
		N	%	N	%	N	%	N	%
**APPROACHING AND HALTERING**	**0**	64	38.8%	63	38.2%	66	40.0%	63	38.2%
	**1**	41	24.8%	50	30.3%	34	20.6%	35	21.2%
	**2**	60	36.4%	52	31.5%	65	39.4%	67	40.6%
**HANDLING**	**0**	80	48.5%	74	44.8%	72	43.6%	72	43.6%
	**1**	68	41.2%	63	38.2%	41	24.8%	67	40.6%
	**2**	17	10.3%	28	17.0%	52	31.5%	26	15.8%
**BUT score**	**0**	64	38.8%	63	38.2%	66	40.0%	63	38.2%
	**1**	11	6.7%	7	4.2%	5	3.0%	7	4.2%
	**2**	34	20.6%	39	23.6%	18	10.9%	26	15.8%
	**3**	40	24.2%	36	21.8%	36	21.8%	47	28.5%
	**4**	16	9.7%	20	12.1%	40	24.2%	22	13.3%
**Classification according to Regulation EC 1/2005**	**Broken**	61	37.0%	40	24.2%	93	56.4%	102	61.8%
	**Unbroken**	104	63.0%	125	75.8%	72	43.6%	63	38.2%

Table S4. Factors affecting eye temperature (ET) of horses from the multivariable model including ET as dependent variable.

Parameter	OR	95% Confidence Interval for OR		P value
		Lower	Upper	
BUT score	1.096	0.998	1.204	0.054
Temperature (°C)	1.062	1.025	1.099	0.001
Lux	1.000	1.000	1.000	0.086
Distance between camera and horse (meters)	0.632	0.521	0.766	<0.001
Age (years)	0.981	0.961	1.002	0.075
Sex (male *vs* female)	0.723	0.508	1.029	0.072

BUT=Broken/Unbroken Test, OR=odds ratio

Table S5. Ordinal logistic regression: factors associated with BUT score

Predictor	OR	95% Confidence Interval for OR		P value
		Lower	Upper	
**HR**	0.948	0.891	1.009	0.093
**RR**	0.849	0.795	0.907	<0.001
**Avoidance distance**	-	-	-	-
**ET**	1.461	0.955	2.235	0.081
**Approach time**	0.977	0.959	0.996	0.015
**Haltering time**	0.978	0.968	0.988	<0.001
**Handling time**	0.967	0.954	0.980	<0.001
**Total time**	0.983	0.978	0.989	<0.001

CI=confidence interval, OR=odds ratio. HR=heart rate, RR= respiratory rate, ET= eye temperature.
- not calculable

Figure S1. Receiver Operating Characteristic (ROC) curve of BUT score for prediction of broken vs unbroken status in horses

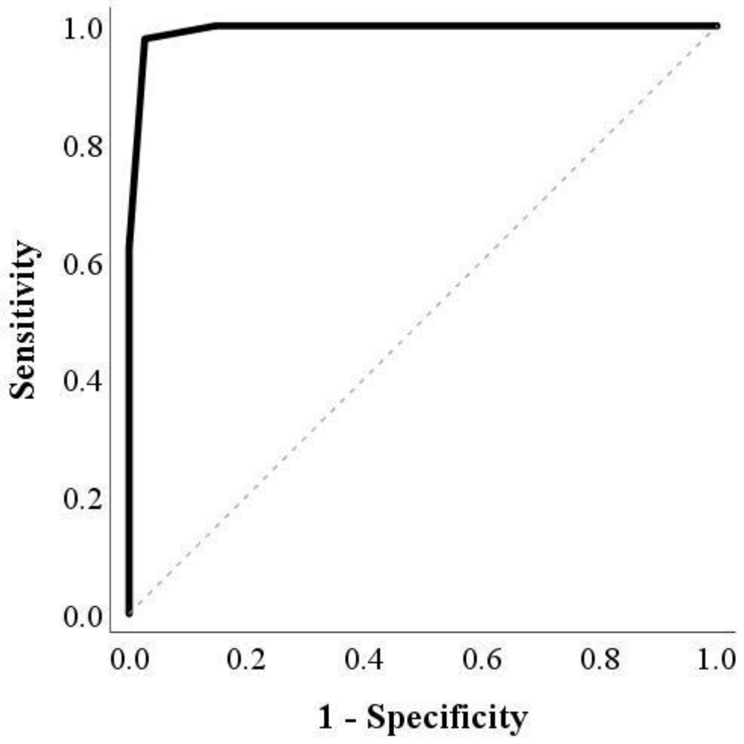

AUC=area under curve, BUT=Broken/Unbroken Test, CI=confidence interval
AUC=0.993, 95% CI=0.984–1.000; P<0.001
Dotted line indicates reference line (AUC=0.50)
